# An oligosaccharyltransferase from *Leishmania donovani* increases the N-glycan occupancy on plant-produced IgG1

**DOI:** 10.3389/fpls.2023.1233666

**Published:** 2023-08-08

**Authors:** Gernot Beihammer, Julia König-Beihammer, Benjamin Kogelmann, Valentina Ruocco, Clemens Grünwald-Gruber, Marc-André D’Aoust, Pierre-Olivier Lavoie, Pooja Saxena, Johannes S. Gach, Herta Steinkellner, Richard Strasser

**Affiliations:** ^1^Department of Applied Genetics and Cell Biology, University of Natural Resources and Life Sciences, Vienna, Austria; ^2^acib - Austrian Centre of Industrial Biotechnology, Vienna, Austria; ^3^Core Facility Mass Spectrometry, University of Natural Resources and Life Sciences, Vienna, Austria; ^4^Medicago Inc., Quebec, QC, Canada; ^5^Division of Infectious Diseases, University of California, Irvine, Irvine, CA, United States

**Keywords:** antibody, glycoprotein, glycosylation, *Nicotiana benthamiana*, recombinant protein

## Abstract

N-Glycosylation of immunoglobulin G1 (IgG1) at the heavy chain Fc domain (Asn297) plays an important role for antibody structure and effector functions. While numerous recombinant IgG1 antibodies have been successfully expressed in plants, they frequently display a considerable amount (up to 50%) of unglycosylated Fc domain. To overcome this limitation, we tested a single-subunit oligosaccharyltransferase from the protozoan *Leishmania donovani* (LdOST) for its ability to improve IgG1 Fc glycosylation. LdOST fused to a fluorescent protein was transiently expressed in *Nicotiana benthamiana* and confocal microscopy confirmed the subcellular location at the endoplasmic reticulum. Transient co-expression of LdOST with two different IgG1 antibodies resulted in a significant increase (up to 97%) of Fc glycosylation while leaving the overall N-glycan composition unmodified, as determined by different mass spectrometry approaches. While biochemical and functional features of “glycosylation improved” antibodies remained unchanged, a slight increase in FcγRIIIa binding and thermal stability was observed. Collectively, our results reveal that LdOST expression is suitable to reduce the heterogeneity of plant-produced antibodies and can contribute to improving their stability and effector functions.

## 1 Introduction

Monoclonal antibodies comprise the most important and fastest-growing class of recombinant biopharmaceuticals used in multiple settings (Walsh and Walsh, 2022). Human immunoglobulin G1 (IgG1) is glycosylated at the conserved asparagine residue 297 (Asn297) in the Fc domain of the heavy chain (HC) and the nature of the attached N-glycan shapes Fc receptor binding and immune-mediated functions (Wang and Ravetch, 2019). Glycosylation is therefore considered an important quality attribute of recombinant antibodies and should be tightly controlled for effective antibody functions, avoidance of unwanted side effects, and development of biosimilars (Reusch and Tejada, 2015).

Plants are increasingly utilized for the production of recombinant biopharmaceuticals (Ruocco and Strasser, 2022; Eidenberger et al., 2023) and successfully used to express different classes of highly effective recombinant antibodies against viruses and human antigens (Chen, 2022; Shanmugaraj et al., 2022). Besides the classical dimeric IgG structure, plants are also able to correctly assemble higher order molecular forms, like dimeric IgA and pentameric IgM (Loos et al., 2014; Dicker et al., 2016; Sun et al., 2021). As shown for IgG antibodies, expression levels of more than 1 g/kg are frequently achieved, making the system economically interesting (Bendandi et al., 2010; Ridgley et al., 2023). Host glycoengineering enables the production of functional IgG antibodies with human-like complex N-glycan structures lacking core fucose residues with increased functional activities (Strasser et al., 2008; Forthal et al., 2010; Zeitlin et al., 2011). However, it has been demonstrated that transient expression of recombinant human IgG1 antibodies in *Nicotiana benthamiana* leaves resulted in the presence of considerable amounts of underglycosylation in the Fc domain (Castilho et al., 2018). Contrary, mammalian cell produced recombinant IgG1 antibodies are typically more than 99% glycosylated at Asn297 (Stadlmann et al., 2008). The observed underglycosylation at the conserved N-glycosylation site increases the heterogeneity (“macroheterogeneity”) of a recombinant IgG1 antibody potentially leading to adverse effect on immune effector functions because non-glycosylated (i.e., both HCs in the assembled antibody lack N-glycans) or hemi-glycosylated (i.e., one HC is unglycosylated, the other HC in the assembled antibody is glycosylated) IgG1 antibodies typically display strongly reduced effector functions (Ha et al., 2011; Ju and Jung, 2014).

In eukaryotes N-glycosylation is typically initiated by the transfer of a preassembled oligosaccharide to specific asparagine residues (Asn-X-Ser/Thr consensus sequence motif) of a translocated polypeptide chain in the lumen of the endoplasmic reticulum (ER). In humans, the reaction is catalyzed by the membrane-embedded multi-protein oligosaccharyltransferase (OST) complex (Shrimal and Gilmore, 2019). While the OST subunit composition and known function of individual protein subunits appear largely conserved in plants (Strasser, 2016), the factors causing the differences in the N-glycosylation efficiency are currently elusive. Here, we performed a detailed characterization of the underglycosylation of different transiently expressed recombinant IgG1 antibodies in glycoengineered *N. benthamiana* and provide a robust approach for efficient N-glycosylation of plant-produced IgG1.

## 2 Materials and methods

### 2.1 IgG1 expression vectors

Construction of expression vectors for pEAQ-Cetuximab (pEAQ-Cx, two independent expression vectors, one for the heavy chain and one for the light chain), pTra-Cetuximab (pTra-Cx, one expression vector carrying two expression cassettes) and magnICON-Cetuximab (magnICON-Cx, two independent expression vectors, one for the heavy chain and one for the light chain) was described recently (Eidenberger et al., 2022). The expression vector for rituximab (Rx) was provided by Medicago (Li et al., 2016) and is referred to as pCAM-Rx (one expression vector carrying two expression cassettes) in this study. For expression in leaf epidermal cells of *N. benthamiana*, the pEAQ-Cx and magnICON-Cx plasmids, were introduced into *Agrobacterium tumefaciens* strain GV3101, while for pTra-Cx *A. tumefaciens* strain GV3101 pMP90RK was used. Mammalian cell produced Rx (Rituxan) was kindly provided by Friedrich Altmann and described previously (Stadlmann et al., 2008).

### 2.2 Cloning of sequences encoding OST subunits

Cloning and expression of LmSTT3D has been described previously (Castilho et al., 2018). For cloning of LdOST, the coding sequence (GenBank: TPP46432.1) was amplified by PCR from synthetic DNA using the forward primer 5’-CTTCCGGCTCGTTTGTCTAGAATG-3’ and the reverse primer 5’-AAAAACCCTGGCGGGATCC-3’. The PCR fragment was digested with *Xba*I/*Bam*HI and subcloned into *Xba*I/*Bam*HI-digested binary expression vector pPT2 (CaMV35S promoter, Strasser et al., 2005) to obtain pPT2-LdOST or p48 (ubiquitin-10 promoter, Hüttner et al., 2014) to obtain p48-LdOST-TAG. In the p48-LdOST-TAG construct a stop codon was included at the end of the LdOST coding sequence to prevent expression of an RFP-fusion protein. To generate p48-LdOST for expression of LdOST-RFP, the LdOST coding sequence without a stop codon was amplified by PCR using the forward primer 5’-CTTCCGGCTCGTTTGTCTAGAATG-3’ and the reverse primer 5’- TATAGGATCCCACCTCACCAAGAGTCCT-3’ and cloned into p48. To generate the human OST4-GFP expression construct, the OST4 coding sequence was amplified by PCR from human cDNA using forward primer 5’-TATATCTAGAATGATCACGGACGTGC-3’ and reverse primer 5’-tataGGATCCTTCCTGCTTCTTGGG-3’, *Xba*I/*Bam*HI-digested and cloned into p47 (ubiquitin-10 promoter, GFP-tag, Hüttner et al., 2014). For ectopic expression, the plasmids were introduced into *A. tumefaciens* strain UIA143 (Strasser et al., 2005).

### 2.3 Plant material and agroinfiltration

*N. benthamiana* plants were grown at 23°C under long-day conditions (i.e., 16 h light/8 h dark). Besides wild-type plants a XT/FT-knockout line (ΔXF KO) was used (manuscript in preparation). Infiltration into leaves of 5-week-old *N. benthamiana* was done as previously described (Göritzer et al., 2017). Briefly, the respective *Agrobacteria* were grown in LB-medium overnight at 29°C. Bacteria were centrifuged, resuspended in infiltration buffer (10 mM MgSO_4_, 10 mM MES and 0.1 mM acetosyringone) and the suspension was used for infiltration. In case of pEAQ-Cx and magnICON-Cx, where light chain and heavy chain are on separate plasmids, *Agrobacteria* suspensions with OD_600_ of 0.15 were infiltrated for the heavy chain while the OD_600_ of *Agrobacteria* suspensions for infiltration of the light chain was 0.1 unless otherwise stated. For pTra-Cx and pCAM-Rx *Agrobacteria* suspensions with OD_600_ of 0.15 were used for infiltration. Binary constructs containing either LdOST or LmSTT3D, respectively, were infiltrated with *Agrobacteria* suspensions with OD_600_ of 0.1.

### 2.4 Confocal microscopy

Analysis of subcellular localization via confocal microscopy was done as previously described (Schoberer et al., 2019). In short, the acquisition of live-cell confocal images was done on a Leica SP5 microscope using an oil immersion objective (Leica 63x/1.4 NA). For GFP excitation an argon laser at 488 nm and for RFP a diode laser at 561 nm was used. For detection a photomultiplier tube (PMT) and Hybrid detectors (HyD) at 500-530 nm and 600-630 nm respectively were employed.

### 2.5 Expression of IgG1 and densitometric analysis

For analysis of underglycosylation via densitometry, IgG1 constructs were infiltrated into leaves of *N. benthamiana* and harvested 3 days post infiltration (dpi). Plant material was mechanically disrupted using a ball mill and 4 µL of ice-cold extraction buffer (0.1 M TRIS, 0.5 M NaCl, 1 mM EDTA, 40 mM ascorbic acid, 2% (w/v), pH 6.8) added to 1 mg of plant material. After removal of cell debris via centrifugation Laemmli buffer was added to the extracts and the samples heated to 95°C for 5 min. Samples were then diluted 1:5 before applying them to SDS-PAGE (12% acrylamide). After electrophoretic separation, proteins were blotted to a nitrocellulose membrane and subsequently detected using an anti-IgG antibody conjugated to horseradish peroxidase (Promega). Proteins were visualized using ECL substrate on a Fusion instrument (Vilber). Densitometric analysis of bands corresponding to glycosylated and unglycosylated IgG1 heavy chain was done using the Evolution-Capt software (Vilber). Background subtraction was done using the rolling ball method of the software.

### 2.6 Mass spectrometric analysis of glycopeptides

For analysis of N-glycans, IgG1 reporters were expressed as described above and enriched via Protein A sepharose as described previously (Castilho et al., 2018). Elution was done using 0.1 M glycine pH 2.8. Following reduction and carbamido-methylation the enriched proteins were subjected to a tryptic in-solution digest (Kolarich and Altmann, 2000) using Trypsin Platinum, Mass Spectrometry Grade (Promega). 10-20 µg protein are routinely digested with 0.5-1.0 µg trypsin for 16 h at 37°C. The resulting glycopeptides were analyzed using an Orbitrap Exploris 480 (Thermo Scientific) and the obtained data analyzed using Skyline Version 22.2 software (MacLean et al., 2010). ANOVA with Tukey *post-hoc* tests were carried out using GraphPad Prism 9.2 software.

### 2.7 Quantification of underglycosylation via mass spectrometry

To quantify the degree of underglycosylation of plant produced IgG1 via mass spectrometry purified proteins were either measured intact or the fraction of unglycosylated heavy chain analyzed from trypsin-digested glycopeptides. For the intact measurement, 1 µg of protein was applied directly to a Waters BioResolve Column (2.1 x 5 mm). An acetonitrile gradient was used ranging from 15% to 80% (v/v) acetonitrile in 0.1% (v/v) formic acid. Gradient time was 15 minutes, flow rate was set to 400 µL/min at 80°C. Detection was performed on a Q-TOF instrument (Agilent Series 6560 LC-IMS-QTOFMS) equipped with the JetStream ESI source in positive mode. Data analysis was done using MassHunter BioConfirm B.08.00. Spectrum deconvolution was performed using MaxEnt. For quantification on the peptide level, glycopeptides were deglycosylated using Protein-N-glycosidase A (PNGase A, Europa Bioproducts Ltd) which leads to deamidation of the glycosylated asparagine residue present in the N-glycosylation site. The resulting mass shift of + 0.984 can be monitored via mass spectrometry. For normalization, a synthetic peptide EEQYNSTYREEQYDSTYR (JPT Peptide Technologies) was used as described (Castilho et al., 2018). Analysis of mass spectrometry data was done using Skyline Version 22.2. ANOVA with Tukey *post-hoc* tests were carried out using GraphPad Prism 9.2 software.

### 2.8 Antigen-binding ELISA

A 20-mer peptide (P20) of the extracellular loop of human CD20 was used as an antigen (Blasco et al., 2007). 1 μg/mL of P20 (diluted in PBS buffer, pH 7.4) was coated (50 µL/well) to 96 well microplates (MicroWell™ MaxiSorp™ Merck) for 16 h at 4°C, then saturated by incubation 100 µL/well with 3% fat free milk powder, dissolved in PBS-T (PBS with 0.05% Tween 20) for 1.5 h at 22°C. Solutions of rituximab diluted in blocking solution and applied to the coated plates in two-fold serial dilutions starting from 1000 µg/mL were then added (50 µL/well) and incubated for 2 h at 22°C to obtain calibration curves. Peroxidase-conjugated goat anti-human gamma chain antibody (Merck) was added at a dilution of 1:5000 and the plates were incubated for a further hour at 22°C. Every step was followed with three-time PBS-T washing. 50 µL/well of the substrate 3,3’,5,5’-tetramethylbenzidine (Merck) was added and plates were incubated for 5 to 10 min. The reaction was stopped with 2 M H_2_SO_4_ and absorbance (λ = 450 nm) with reference to 620 nm was measured with an ELISA reader (Tecan Spark® spectrophotometer). The binding curve was generated using GraphPad Prism 9.2 software.

### 2.9 Fcγ receptor binding by flow cytometry

FcγRIIIa (CD16a; F158 allotype) expressing TZM-bl cells were used to assess the binding affinity of Rx variants. Flow cytometry was carried out exactly as described (Forthal et al., 2010). The binding curves were generated by plotting the mean fluorescence intensity of positive cells indicating receptor binding as a function of antibody concentration. Unspecific binding to wild-type cells was subtracted from binding to FcγRIIIa-expressing cells. Each antibody concentration was run in duplicate. Binding experiments were repeated three times.

### 2.10 SPR

*In vitro* binding experiments of Rx variants to the extracellular domain (amino acids 17-208) of FcγRIIIa/CD16a was performed using a Biacore T200 (Cytiva). First the sensor chip surface was captured with anti-His antibody with the His Capture Kit (Cytiva) to a CM5 chip as described in the manufacturers’ protocol. The capturing of anti-His antibody reached 32000 RU. Secondly, immobilization of the His-tagged human FcγRIIIa (F158 allotype) or FcγRIIIa (V158 allotype) (AcroBiosystems) on the chip surface was performed for 60s with a concentration of 1 μg/mL and a flow rate of 10 μL/min in HEPES-EP running buffer. 40 RU units were achieved. The immobilization step was previously optimized in order to avoid avidity effects using lower concentration of FcγRIIIa and shorter times. Flow cell 2 remained unmodified and served as a reference cell for the subtraction of systematic instrument noise and drift. Rx binding curves were generated in multi-cycle kinetic experiments at seven different concentrations ranging from 7.8 to 500 nM with 60 seconds association and 60 seconds dissociation time at a flow rate of 10 μL/min. After each run, surface regeneration was accomplished using 10 mM glycine, pH 1.7, for 120 seconds at a flow rate of 30 μL/min. Binding affinities (K_D_) were calculated with Biacore T2 Evaluation software using a 1:1 binding in kinetics (Rx, Rx + LdOST, Rx + LmSTT3D) or steady state (Rituxan). All experiments were repeated as three independent kinetic runs.

### 2.11 DSF

Protein stability measurements were carried out by differential scanning fluorimetry (DSF) using the CFX96 Real-Time PCR Detection System (Bio-Rad) with a final dilution of 1:500 of the SYPRO Orange dye (Molecular Probes). Fluorescence of a 25 μL sample (final concentration 0.4 mg/mL) in PBS was recorded from 10 - 95°C (0.5°C increments, 10 seconds hold per step) using the FRET channel. The thermograms, both the normalized relative fluorescence units (RFU) and the normalized derivative of relative fluorescence units (d(RFU)/dT) with respect to temperature (T) were recorded and compared. The peaks of the d(RFU)/dT-T thermogram are regarded as the melting temperatures (Tm) of the corresponding protein. Triplicate measurements were performed for each protein. Consistent with previous literature, the first peak of the mAb was attributed to the unfolded Fc fragment, while the second transition was interpreted as the melting of the Fab fragment.

## 3 Results

### 3.1 Expression conditions have a minor effect on N-glycosylation site occupancy

A genome edited β1,2-xylosyltransferase and core α1,3-fucosyltransferase *N. benthamiana* knockout line devoid of xylose and fucose carrying N-glycans (ΔXF KO), was used as expression host (manuscript in preparation). Two therapeutic IgG1 antibodies, namely rituximab (Rx) carrying only the conserved N-glycosylation site Asn297 and cetuximab (Cx) which has an additional N-glycosylation site in the Fab domain of the HC were characterized (Castilho et al., 2015; Li et al., 2016; Eidenberger et al., 2022). First, antibodies were transiently expressed under different conditions, including the use of various vector backbones (i.e. magnICON, pEAQ, pCAM, pTra), either as single genes or in tandem repeats, different bacterial concentrations for agroinfiltration and harvesting at diverse time points. Recombinant antibody expression was monitored by immunoblotting of total soluble protein extracts, with focus on the two HC associated bands, representing the glycosylated (~ 53 kDa band) and the underglycosylated HC (~ 50 kDa, **Supplemental Figure S1**). Densitometric analyses revealed significant amounts of underglycosylated HC in all settings, varying from 15-30% for Rx and 30-55% for Cx. These results are in line with previous observations (Castilho et al., 2018; Eidenberger et al., 2022) and demonstrate that changing expression conditions only had a minor impact on the N-glycosylation efficiency.

### 3.2 LdOST from the protist *Leishmania donovani* is retained in the ER of plant cells

Recently we characterized LmSTT3D, a single-subunit OST from *Leishmania major* that increases the N-glycosylation efficiencies of plant-produced recombinant glycoproteins (Castilho et al., 2018). GFP tagged LmSTT3D transiently expressed in *N. benthamiana* was located in the ER and displayed punctate structures that resemble Golgi bodies as reported previously (Castilho et al., 2018). A more detailed analysis by confocal microscopy revealed that LmSTT3D-GFP exhibits unusual aggregate-like subcellular structures (**Supplemental Figure S2A**) and can alter the N-glycan profile of co-expressed recombinant antibodies towards mannosidic structures (**Supplemental Figure S2B**). While this negative impact on N-glycan processing was only occasionally observed, these findings indicate a possible interference of LmSTT3D with the plant endogenous glycosylation pathway that should be avoided for the robust production of homogenous recombinant glycoproteins.

To overcome this potential limitation of LmSTT3D, sequence databases of protists were searched for alternative single-subunit OSTs. From the obtained hits we selected the single-subunit OST from *Leishmania donovani*. While this protein termed LdOST displayed less than 80% amino acid sequence identity to LmSTT3D, crucial amino acid residues in the substrate binding region appear all conserved in LdOST and the predicted membrane protein topology is similar to LmSTT3D. This makes LdOST a potential single-subunit OST candidate to overcome IgG1 underglycosylation in plants (**Supplemental Figures S3**, **S4**).

**Figure 1:**
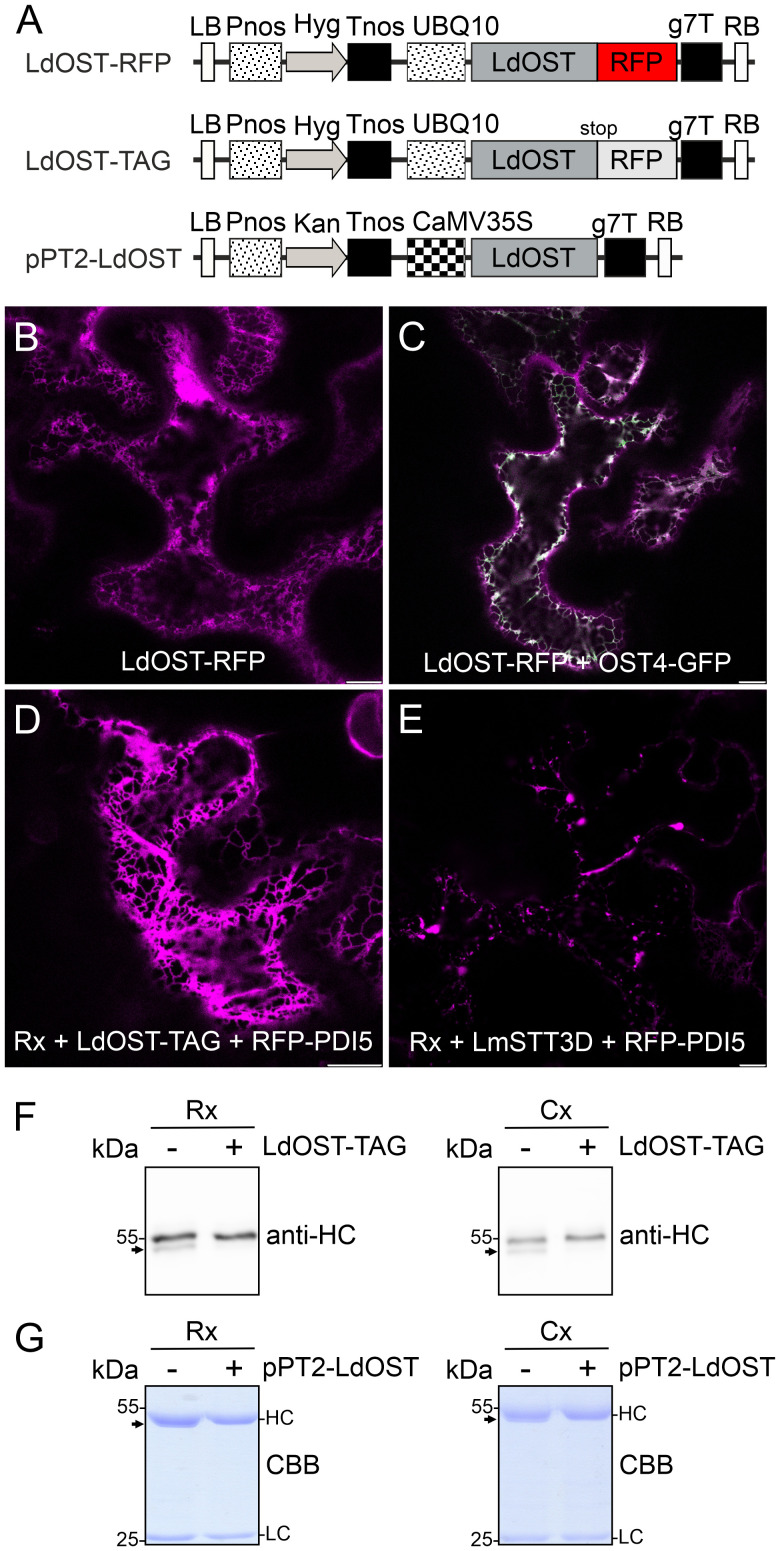
LdOST-RFP accumulates in the ER and increases the N-glycan occupancy on the IgG HC. **(A)** Schematic representation of the LdOST expression vectors. LB, left border; Pnos, nopaline synthase gene promoter; Hyg, hygromycin B phosphotransferase gene; Tnos, nopaline synthase gene terminator; UBQ10, *A*. *thaliana* ubiquitin-10 promoter; LdOST, *L. donovani* OST subunit open reading frame; RFP, red fluorescent protein; g7T, *Agrobacterium* gene 7 terminator; RB, right border. CaMV35S, cauliflower mosaic virus 35S promoter; Kan, neomycin phosphotransferase II gene. LdOST-TAG has a stop-codon that prevents fusion to RFP. **(B)** Confocal microscopy image of LdOST-RFP (magenta) expressed in wild-type *N. benthamiana* leaf epidermal cells. **(C)** Representative wild-type cell co-expressing LdOST-RFP (magenta) and the ER-marker OST4-GFP (green, co-localization in white). **(D)** pCAM-Rx (Rx) was co-expressed with LdOST-TAG and the ER marker RFP-PDI5 (magenta) in wild-type *N. benthamiana* leaf epidermal cells. **(E)** pCAM-Rx (Rx) was co-expressed with LmSTT3D and RFP-PDI5 (magenta) in wild-type *N. benthamiana* leaf epidermal cells. Scale bars = 10 µm. **(F)** pCAM-Rx (Rx) or cetuximab (Cx) antibodies were co-infiltrated with LdOST-TAG. Proteins were extracted from *N. benthamiana* ΔXF KO line 3 dpi and subjected to SDS-PAGE and immunoblotting using anti-IgG (anti-HC) antibodies. **(G)** SDS-PAGE and Coomassie Brilliant Blue (CBB) staining of purified Rx and Cx co-expressed with pPT2-LdOST in *N. benthamiana* ΔXF KO. The arrows indicate the migration position of the unglycosylated HC band.

We generated an LdOST-RFP fusion protein (**Figure 1A**), transiently expressed it in *N. benthamiana* and analyzed the subcellular localisation in leaf epidermal cells using confocal microscopy. In contrast to LmSTT3D-GFP (**Supplemental Figure S2A**), LdOST-RFP displayed exclusively ER labelling and no aberrant cellular structures that are indicative of aggregate formation or mislocalization. Co-localization with a GFP-tagged ER-marker (OST4-GFP) (**Figure 1C**) confirmed the subcellular localization (**Figure 1B**). When an untagged LdOST variant was co-expressed with pCAM-Rx and the ER-marker RFP-PDI5 (Shin et al., 2021), a typical ER labelling was observed for the ER marker suggesting that LdOST has no obvious adverse effect on the ER morphology (**Figure 1D**). By contrast, Rx plus LmSTT3D-GFP and RFP-PDI5 expression resulted in the formation of an aberrant ER with accumulation of the ER marker in punctuate structures (**Figure 1E**).

### 3.3 LdOST is a functional single-subunit OST that improves N-glycosylation of IgG1 antibodies

**Figure 2:**
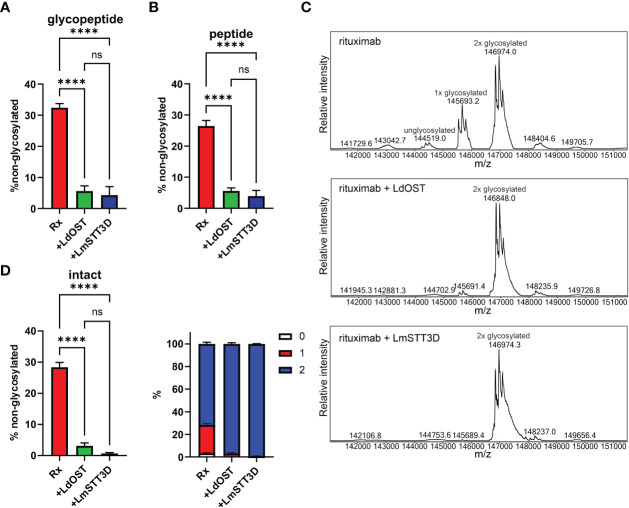
Quantification of the underglycosylation of rituximab (Rx). **(A)** % of non-glycosylated Asn297 in the HC of Rx. pCAM-Rx was expressed alone (Rx) (OD_600_ = 0.15) or in combination with LdOST-TAG (OD_600_ = 0.1) and LmSTT3D (OD_600_ = 0.1), respectively, in *N. benthamiana* ΔXF KO, purified and subjected to trypsin digestion. The tryptic peptides were analyzed by LC-ESI-MS. Error bars indicate mean ± SD (n = 3, “****” P < 0.0001 according to ANOVA). **(B)** Purified Rx samples were digested with trypsin, deglycosylated and analyzed by LC-ESI-MS. The amounts of deglycosylated and non-glycosylated peptides were obtained by comparison to an internal standard peptide treated in the same way. Error bars indicate mean ± SD (n = 3, “****” P < 0.0001 according to ANOVA). **(C)** The N-glycan site occupancy of fully assembled intact IgG was determined using LC-ESI-MS. The peaks corresponding to unglycosylated, hemi-glycosylated (1x glycosylated) and fully glycosylated (2x glycosylated) are highlighted. Multiple peaks represent different glycoforms and variations in the clipping of C-terminal lysine. **(D)** Quantification of the peaks from **(C)** Error bars indicate mean ± SD (n = 3, “****” P < 0.0001 according to ANOVA).

**Figure 3:**
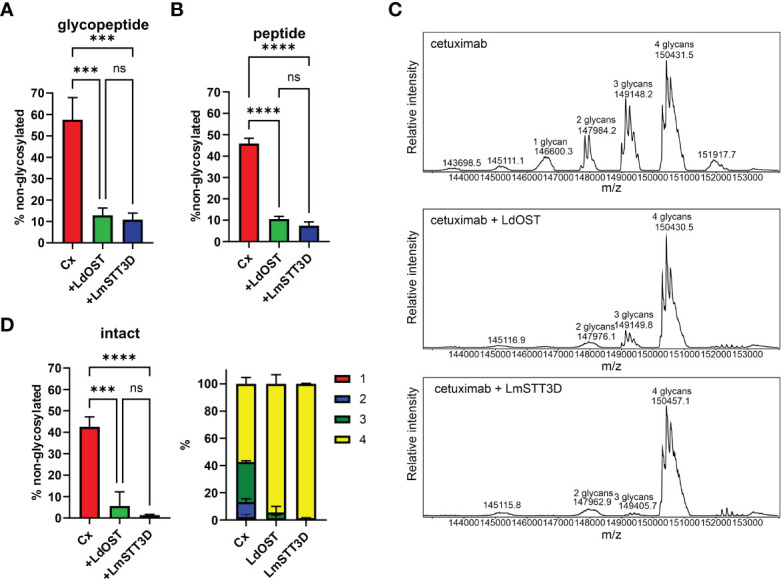
Quantification of the underglycosylation of cetuximab (Cx). **(A)** % of non-glycosylated Asn297 in the HC of Cx. Cx was expressed alone (HC OD_600_ = 0.15; LC OD_600_ = 0.1) or in combination with LdOST-TAG (OD_600_ = 0.1) and LmSTT3D (OD_600_ = 0.1), respectively, in *N. benthamiana* ΔXF KO, purified and subjected to trypsin digestion. The tryptic peptides were analyzed by LC-ESI-MS. Error bars indicate mean ± SD (n = 3, "***" P<0.001 according to ANOVA). **(B)** Purified Cx samples were digested with trypsin, deglycosylated and analyzed by LC-ESI-MS. The amounts of deglycosylated and non-glycosylated peptides were obtained by comparison to an internal standard peptide treated in the same way. Error bars indicate mean ± SD (n = 3, “****” P < 0.0001 according to ANOVA). **(C)** The N-glycan site occupancy of fully assembled intact IgG was determined using LC-ESI-MS. The peaks corresponding to assembled antibody carrying 1 to 4 N-glycans highlighted. Multiple peaks represent different glycoforms and variations in the clipping of C-terminal lysine. **(D)** Quantification of the peaks from C. Error bars indicate mean ± SD (n = 3, "***" P < 0.001, "****" P < 0.0001 according to ANOVA).

Next, we transiently co-expressed untagged LdOST with pCAM-Rx and Cx, respectively, in *N. benthamiana* ΔXF KO. In crude leaf extracts and on affinity-purified antibodies, the faster migrating HC band at approximately 50 kDa, representing the underglycosylated HC, disappeared in the presence of LdOST suggesting the expression of an active OST subunit (**Figures 1F, G**). Three different MS-based methods were applied to exactly quantify glycosylated versus non-glycosylated Fc of purified antibodies. First, Rx and Cx antibodies were subjected to tryptic digestion and the respective Asn297 containing polypeptides analyzed by LC-ESI-MS (**Figures 2A**, **3A**). Second, N-glycans were released from the glycopeptides by digestion with PNGase A and the mixture of deglycosylated (EEQYQSTYR) and unglycosylated (EEQY**N**STYR) peptides was examined by LC-ESI-MS. A peptide harbouring both the unglycosylated and deglycosylated (N to Q conversion) amino acid sequence motif was used as an internal standard for quantification and processed in the same way as the samples (**Figures 2B**, **3B**) (Castilho et al., 2018). Finally, intact fully assembled IgG1 was analyzed by MS (**Figures 2C, D**, **3C, D**).

Collectively, in the absence of any OST subunit, both antibodies displayed substantial amounts of unglycosylated HC-Fc with clear differences between Rx and Cx (26% and 46% non-glycosylated, respectively) (**Figures 2B**, **3B**). LmSTT3D and LdOST were both able to significantly reduce the underglycosylation at site Asn297 of both antibodies (down to 3 and 10%, respectively). Intact MS-measurement of the assembled antibodies showed considerable amounts of non- and hemi-glycosylated Rx (together 28%) (**Figure 2C**) and Cx (together 43%) (**Figure 3C**). These values were significantly reduced by OST subunit co-expression with up to 97% of the assembled antibodies being fully glycosylated. Interestingly, under all tested conditions, the N-glycan occupancy at the Cx Fab N-glycosylation site was more than 99% (**Supplemental Figure S5**).

**Figure 4:**
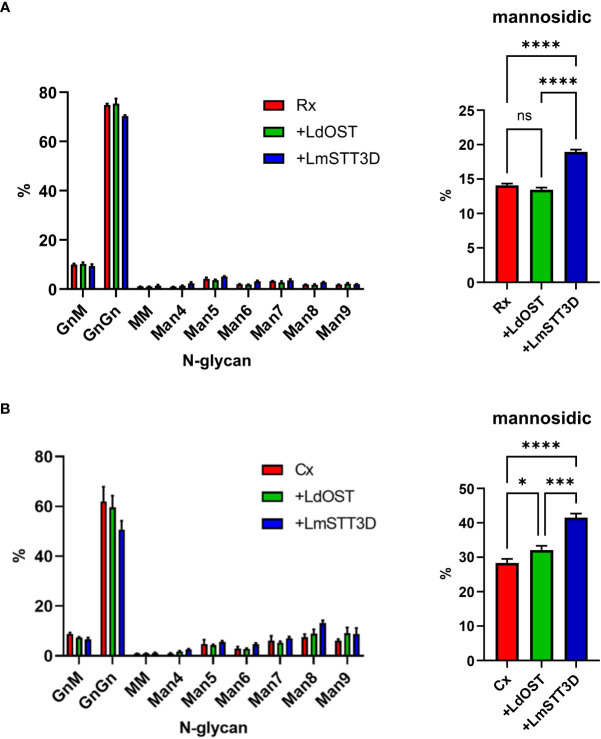
Quantification of the N-glycan composition at Asn297 of rituximab and cetuximab expressed in the *N. benthamiana* ΔXF KO line. **(A)** Glycoforms of rituximab (Rx). **(B)** Glycoforms of cetuximab (Cx). Bars represent the relative abundance (%) of glycoforms present at Asn297 in the Fc domain as determined by LC-ESI-MS of the glycopeptide EEQYNSTYR. For abbreviations of N-glycans the ProGlycAn system was used (https://www.proglycan.com/). Mannosidic N-glycans are the sum of Man4 to Man9. Error bars indicate mean ± SD (n = 3, “ns” not significant, “*” P < 0.05, “***” P < 0.001 “****” P < 0.0001 according to ANOVA).

Expression of a single-subunit OST might directly or indirectly affect the N-glycan composition on the antibody. The direct effect could be caused by the transfer of an incompletely assembled oligosaccharide precursor (Man_5_GlcNAc_2_ to Glc_2_Man_9_GlcNAc_2_ intermediates) (Kelleher and Gilmore, 2006). The indirect effect could be linked to changes in the secretory pathway which may alter the organisation of glycosyltransferases in the Golgi apparatus and thus N-glycan processing. Therefore, the N-glycan composition was determined by LC-ESI-MS. While, the N-glycan profile did not change by overexpression of LdOST, LmSTT3D co-expression resulted in a small but significant increase in oligomannosidic N-glycans (**Figure 4**; **Supplemental Figures S6**, **S7**). This increase was also observed for the Cx Fab N-glycans (**Supplemental Figures S5**, **S8**) and is consistent with the observed tendency of LmSTT3D to interfere with ER-morphology.

### 3.4 Improved N-glycan occupancy on rituximab increases the binding affinity to FcγRIIIa receptors

**Figure 5:**
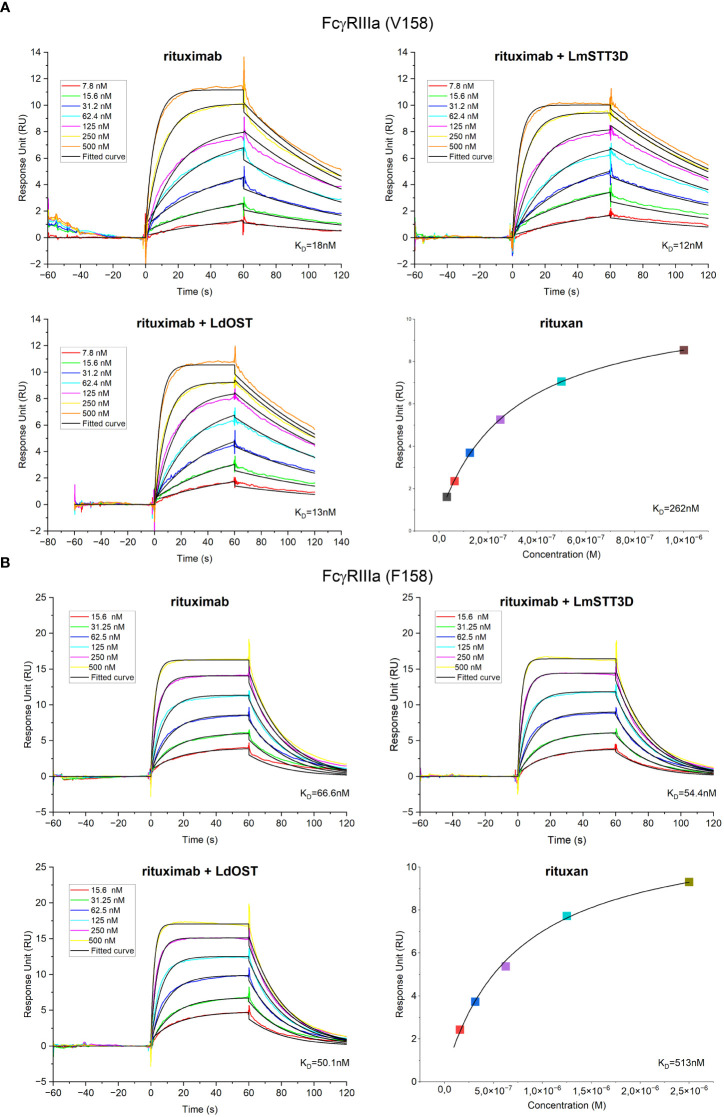
Receptor binding characteristics of rituximab (Rx). K_D_ values were obtained by SPR spectroscopy in multi-cycle kinetic experiments from three independent measurements for **(A)** FcγRIIIa (V158) and **(B)** FcγRIIIa (F158). Sensorgrams exhibit seven different concentrations ranging from 7.8 to 500 nM for FcγRIIIa (V158) and from 15.6 to 500 nM for FcγRIIIa (F158). Binding of the control rituxan was analyzed in steady state.

Having established that LdOST is functional, we further examined the impact of LdOST expression on Rx function. An antigen binding ELISA showed equal binding for the three tested Rx variants - Rx alone, Rx co-expressed with LdOST and Rx co-expressed with LmSTT3D, respectively (**Supplemental Figure S9**). In accordance, in a cell-based assay, Rx co-expressed with LdOST or with LmSTT3D displayed comparable binding to cells expressing the low affinity FcγRIIIa (F158 allotype) (**Supplemental Figure S10**). To analyze the binding kinetics in more detail and determine the binding affinities, we carried out SPR with the ectodomains of FcγRIIIa (F158) and FcγRIIIa (V158) (**Figure 5**). Rx co-expressed with LdOST and LmSTT3D, respectively, displayed reduced K_D_ values with both receptors indicating that improved N-glycosylation site occupancy increases the binding affinity to the receptor (**Supplemental Table S1**). The K_D_ of the mammalian cell-derived Rx (rituxan) was at least 10-fold higher being consistent with the negative impact of the core fucose on FcγRIIIa binding (Shields et al., 2002; Ferrara et al., 2011). Finally, we compared the thermal stability of the antibodies. While all plant-produced Rx variants displayed melting points that were comparable to rituxan, the Rx co-expressed with LdOST variant exhibited a thermal unfolding transition of the CH2 domain at a slightly higher temperature (+ 1°C) than Rx alone (**Table 1**; **Supplemental Figure S11**).

**Table 1:** Thermal stability (DSF).

Antibody	First peak (°C)	Second peak (°C)
Rituxan	68.8 ± 0.4	74.7 ± 0.3
Rituximab	68.8 ± 0.3	75.0 ± 0.0
Rituximab (LmSTT3D)	69.0 ± 0.5	75.2 ± 0.3
Rituximab (LdOST)	69.8 ± 0.4	75.2 ± 0.3

## 4 Discussion

Plant-produced recombinant IgG1 displays considerable amounts of HC that is non-glycosylated at the conserved Asn297 site. This has been shown for transiently produced IgG1 in *N. benthamiana* leaves (Castilho et al., 2018; Jansing et al., 2019; Eidenberger et al., 2022) and for stable expression of antibodies derived from different tissues and species (Rademacher et al., 2008; Vamvaka et al., 2016). In contrast, mammalian cell-derived recombinant IgG1 is fully glycosylated at this site (Stadlmann et al., 2008). As previously reported, the magnICON expression vector-derived antibody displayed reduced glycosylation likely due to high expression levels and a subsequent overload of the endogenous plant OST machinery (Eidenberger et al., 2022). Our attempts to elucidate other factors that cause inefficient Fc glycosylation did not result in noticeable reduced or enhanced N-glycosylation efficiency suggesting that an unknown OST complex intrinsic feature is another major contributor for IgG1 underglycosylation. While most of the OST subunits, including the catalytic STT3 subunit, are present in plants and play a similar role in the N-glycosylation process (Koiwa et al., 2003; Lerouxel et al., 2005; Farid et al., 2013; Jeong et al., 2018), the molecular differences in distinct OST subunits or complex composition that affect substrate recognition or transfer of the oligosaccharide in plants are currently unknown. Since the Fab N-glycosylation site on the Cx HC is almost fully glycosylated, the limitation of the plant OST complex is very specific for distinct sites like Asn297 and is likely caused by the local amino acid environment adjacent to the N-glycosylation site (Murray et al., 2015). While the underglycosylation of highly expressed antibodies could potentially be counterbalanced by overexpression of the whole complex or individual plant OST subunits, expression of non-plant subunits (like LdOST or LmSTT3D) or protein engineering of plant OST subunits can provide solutions to overcome the plant OST complex intrinsic underglycosylation.

In previous studies, we utilized transient expression of LmSTT3D to increase the N-glycan occupancy on different recombinant glycoproteins expressed in plants (Castilho et al., 2018; Göritzer et al., 2020). Nevertheless, we occasionally observed unwanted side effects, like increased amounts of incompletely processed N-glycan structures on recombinant glycoproteins. This suggests an interference with other glycosylation related pathways and cellular processes that is likely related to mislocalization of LmSTT3D to the Golgi apparatus and other vesicular structures and an alteration of ER-derived structures (Castilho et al., 2018). In this regard, LdOST appears superior as it displayed only ER localisation and its co-expression with recombinant antibodies did not lead to unwanted alterations of the overall N-glycan profile (**Supplemental Figure S12**).

Importantly, biochemical and functional features of LdOST and LmSTT3D co-expression did not negatively impact biochemical and functional activities of the antibodies. In fact, some obvious glycosylation dependent activities, like FcγRIIIa binding affinities, were increased. These results are in line with previous studies that report decreased FcγRIIIa binding of hemi-glycosylated IgG1 (Ha et al., 2011). Also, the melting temperatures of Rx co-expressed with LdOST or with LmSTT3D were slightly higher than the one for Rx which is consistent with a reduced thermal stability of non-glycosylated IgG1 that mainly affects the CH2 domain (Garber and Demarest, 2007; Zheng et al., 2011; Hristodorov et al., 2013).

Augmentation of the N-glycosylation efficiency is not only relevant for IgG1 antibodies but also improves the formation of dimeric IgA antibodies (Göritzer et al., 2020) and is used together with chaperone co-expression as a technology to promote N-glycosylation-directed folding pathways in plants (Margolin et al., 2022; Margolin et al., 2023). We anticipate that LdOST is a useful single-subunit OST that can be broadly applied to overcome constraints in the plant production platform. Taken together, we exploited a novel glycoengineering tool that reduces the heterogeneity of plant made recombinant IgG1 antibodies without any adverse effect on functional properties. Given the high amounts of underglycosylation on plant-produced IgG1 and the noticeable effect of incompletely glycosylated IgG1 on FcγRIIIa binding, it is advisable to use this glycoengineering approach for recombinant IgG1 production in plants to optimize effector functions.

## Data availability statement

The data presented in the study are deposited in the PRIDE repository (Perez-Riverol et al., 2022), accession number PXD043623.

## Author contributions

GB, JK-B, BK, VR, CG-G, and JG conducted the experiments. GB, VR, CG-G, M-AD’A, P-OL, PS, HS, and RS analyzed the results. RS conceptualized the study and wrote the paper with support from all authors. All authors contributed to the article and approved the submitted version.
