# Supplementary material for: An oligosaccharyltransferase from Leishmania donovani increases the N-glycan occupancy on plant-produced IgG1
Source: Front Plant Sci. 2023 Aug 8;14:1233666. doi: 10.3389/fpls.2023.1233666 (PMC10442823; doi:10.3389/fpls.2023.1233666)
Supplement: Supplementary file 1 [file DataSheet_1.pdf]

Supplementary Material

An oligosaccharyltransferase from *Leishmania donovani* increases the N-glycan occupancy on plant-produced IgG1

Gernot Beihammer[1,2], Julia König-Beihammer[1], Benjamin Kogelmann[1,2], Valentina Ruocco[1], Clemens Grünwald-Gruber[3], Marc-André D'Aoust[4], Pierre-Olivier Lavoie[4], Pooja Saxena[4], Johannes S. Gach[5], Herta Steinkellner[1], Richard Strasser[1]*

[1]Department of Applied Genetics and Cell Biology, University of Natural Resources and Life Sciences, Vienna, Austria

[2]acib - Austrian Centre of Industrial Biotechnology, Muthgasse 18, 1190 Vienna, Austria.

[3]Core Facility Mass Spectrometry, University of Natural Resources and Life Sciences, Vienna, Austria

[4]Medicago Inc., Quebec, QC, Canada

[5]University of California, Irvine, Division of Infectious Diseases, Irvine, CA,

*Correspondence:

Richard Strasser

Email: richard.strasser@boku.ac.at

Keywords: antibody, glycoprotein, glycosylation, *Nicotiana benthamiana*, recombinant protein

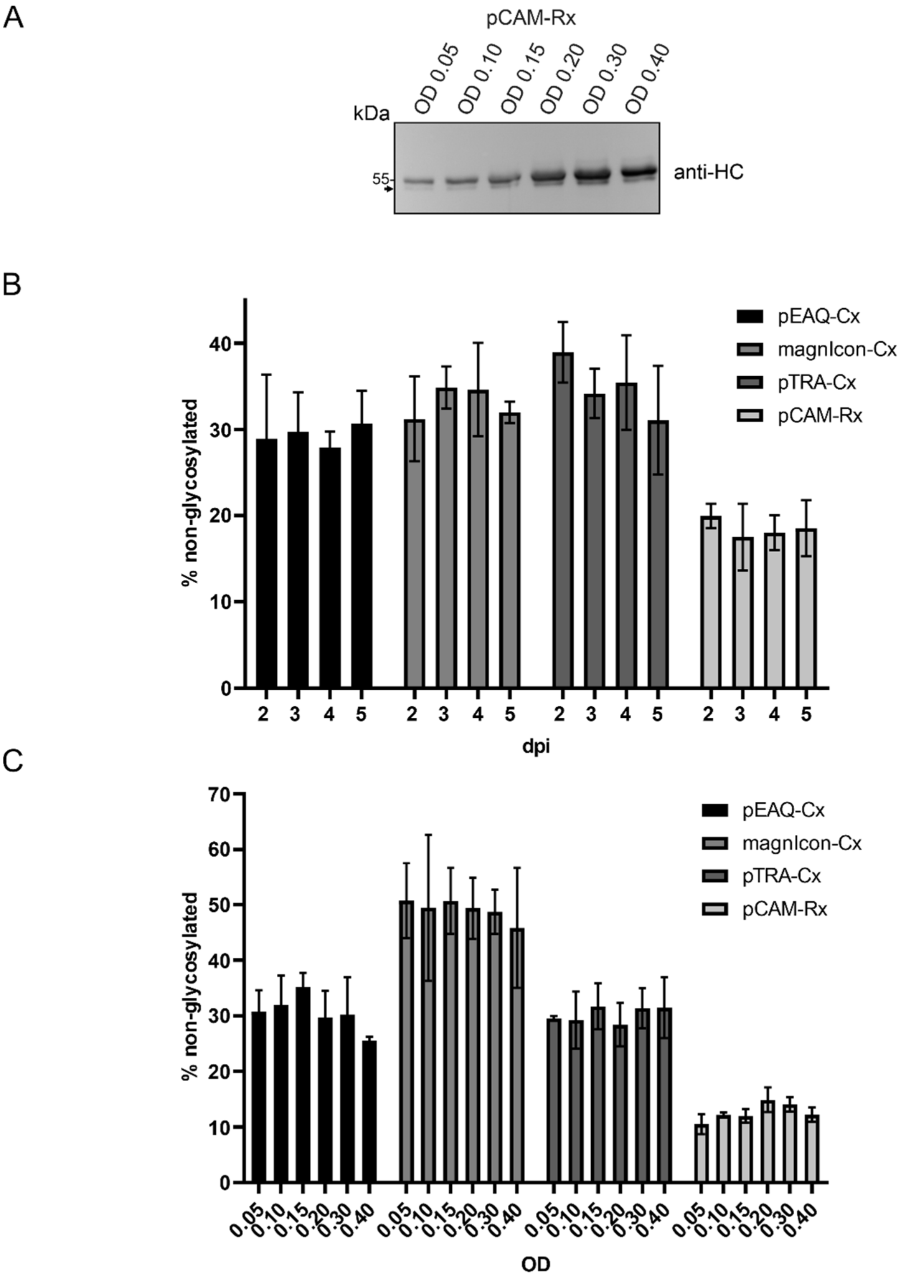

Figure S1. Quantification of rituximab (Rx) and cetuximab (Cx) underglycosylation at different expression conditions. Agrobacteria carrying the indicated constructs were infiltrated into leaves of ΔXF KO plants. Crude protein extracts were mixed with Laemmli buffer, separated by SDS-PAGE and subjected to immunoblotting using anti-heavy chain (HC) antibodies. Quantification was done by densitometric analysis of the immunoblot bands that correspond to glycosylated and unglycosylated HC. (**A**) Representative image of an immunoblot detected with an anti-human IgG antibody (H+L - HRP conjugate, Promega). The unglycosylated HC band is marked by an arrow (**B**) Effect of the harvesting time (in days post infiltration - dpi). (**C**) Effect of OD_{600} used for infiltration of both the HC and light chain (LC). The same OD_{600} was used for HC and LC expression. Error bars indicate mean ± SD ($n \geq 3$) from protein extracts obtained by independent infiltrations of plants.

A

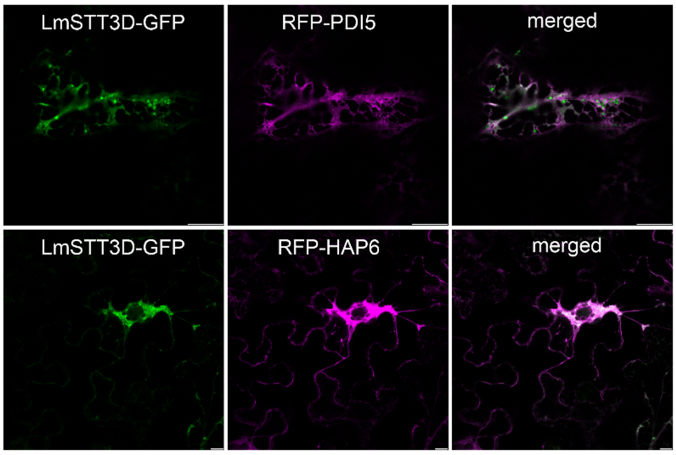

B

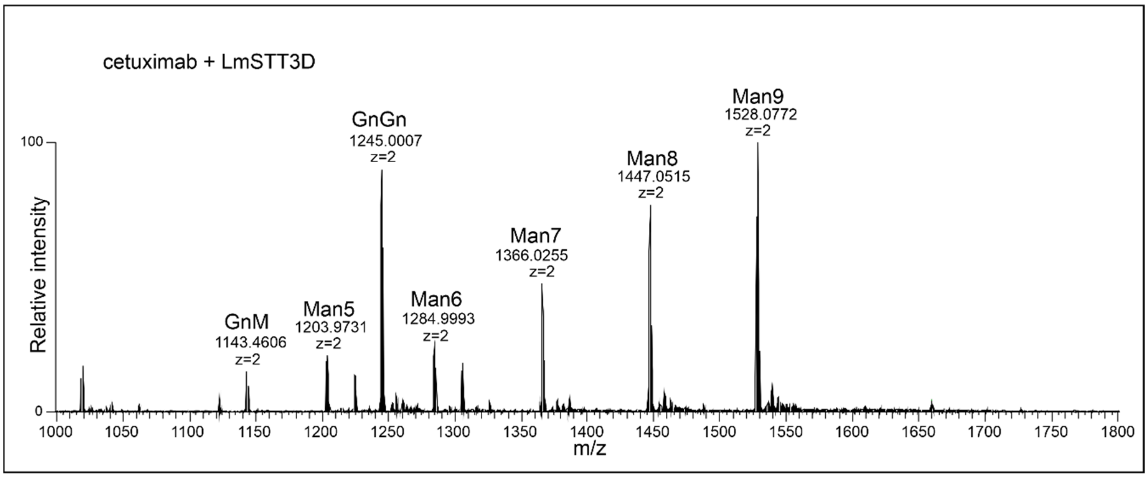

Figure S2. Problems observed with **LmSTT3D-GFP expression.** (**A**) LmSTT3D-GFP was co-expressed with the soluble ER marker RFP-PDI5 (Shin et al., 2021) or the membrane-anchored ER marker RFP-HAP6 (p117-HAP6) in leaves of *N. benthamiana* wild-type and images of fluorescent proteins were acquired by confocal microscopy 2 dpi. Merged images show the overlay of the GFP/RFP signals in white. Scale bars = 10 µm. (**B**) MS-spectrum showing an increase of oligomannosidic N-glycans in the presence of LmSTT3D. Cetuximab was co-expressed with LmSTT3D-GFP in ΔXF KO, purified from protein extracts of leaves harvested 4 dpi and subjected to trypsin digestion and LC-ESI-MS. Peaks corresponding to glycosylated peptide EEQYNSTYR carrying Asn297 are assigned in the spectrum. Nomenclature according to the ProGlycAn system: www.proglycan.com).

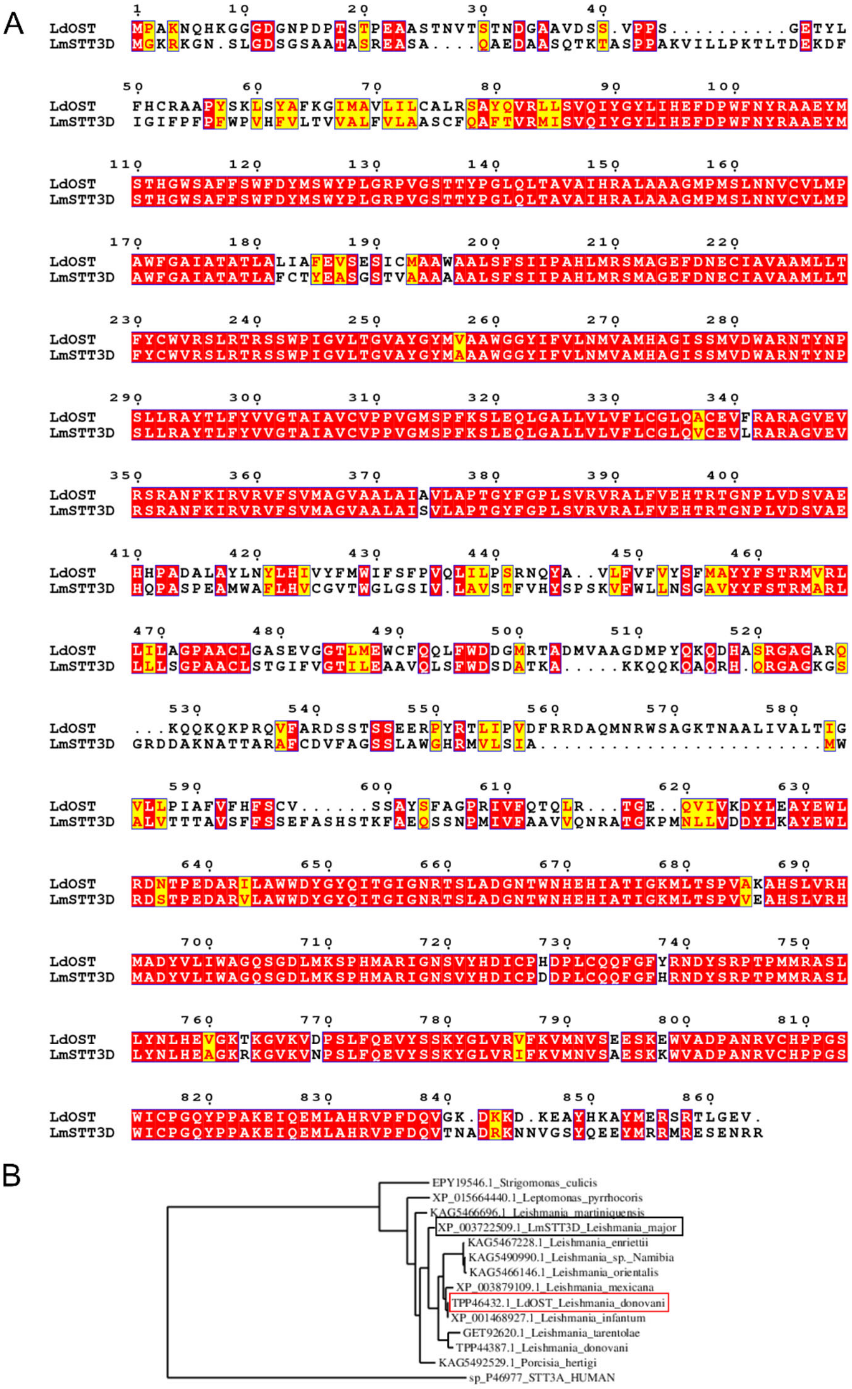

Figure S3. Alignment and phylogenetic tree of different OST sequences. (**A**) Alignment of amino acid sequences of LdOST (TPP46432.1) and LmSTT3D (XP_003722509.1) was done using T-Coffee (https://tcoffee.crg.eu/apps/tcoffee/do:expresso) and visualized using ESPript 3.0 (https://espript.ibcp.fr/ESPript/cgi-bin/ESPript.cgi). While numerous amino acid sequences in transmembrane domains and the luminal region at the C-terminus are fully conserved (red background), the large loop closer to the C-terminal end that faces the cytosol displays a high sequence variation. (**B**) Phylogenetic tree generated using Phylogeny.fr (https://www.phylogeny.fr/phylogeny.cgi) of different sequences retrieved by the BLASTP search.

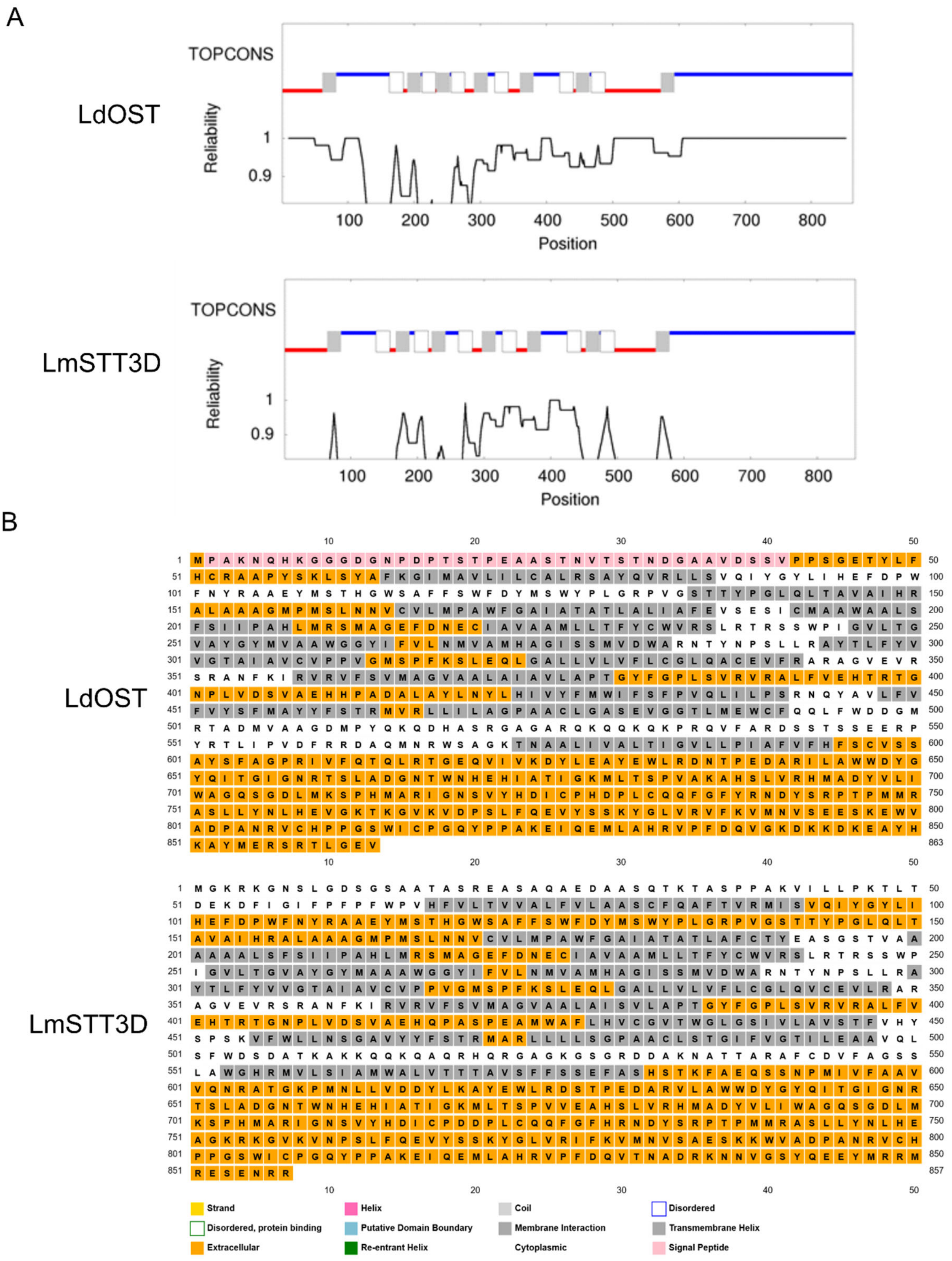

Figure S4. Comparison of the membrane protein topology prediction for LdOST and LmSTT3D. (A) Topcons prediction (https://topcons.cbr.su.se/). **(B)** PSIPRED-Memsat prediction (http://bioinf.cs.ucl.ac.uk/psipred/&uuid=ca774416-9c14-11ed-95cd-00163e100d53). Based on these predictions the two proteins share a similar protein topology.

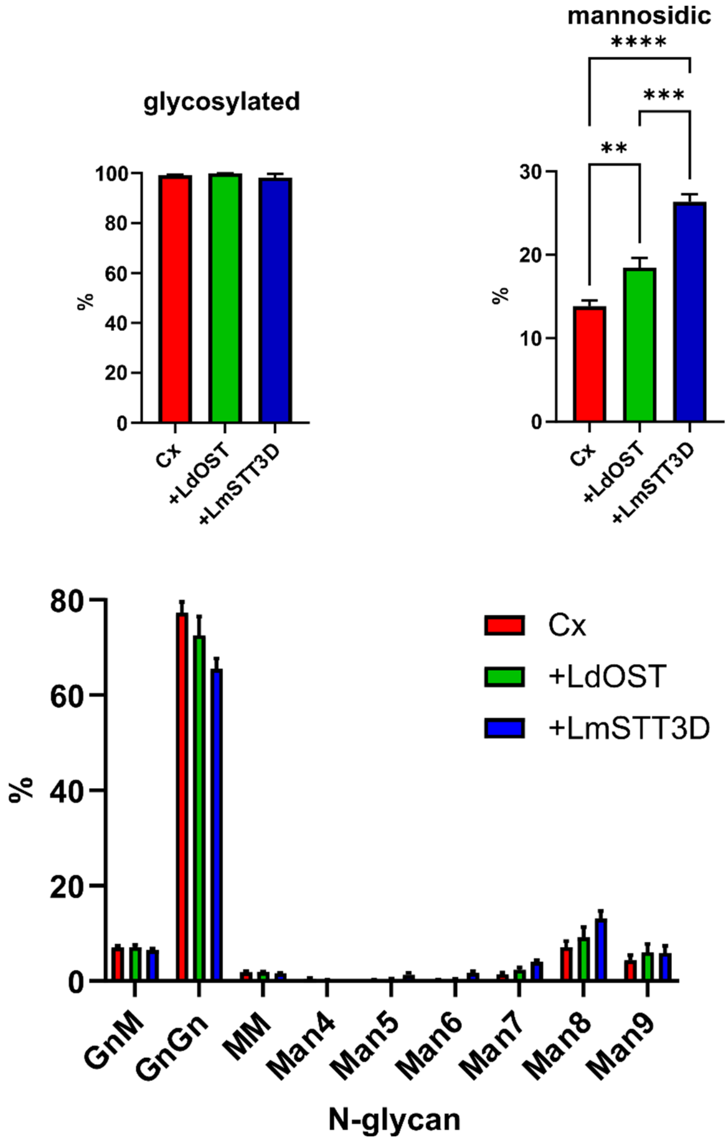

Figure S5. Quantification of the cetuximab (Cx) Fab N-glycosylation site occupancy and glycoforms. Bars represent the relative abundance (%) of glycoforms present at Asn88 in the variable domain of the Cx HC as determined by LC-ESI-MS of the glycopeptide MNSLQSNDTAIYYCAR. Cx was expressed in the *N. benthamiana* ΔXF KO line. For abbreviations of N-glycans the ProGlycAn system was used (https://www.proglycan.com/). Mannosidic N-glycans are the sum of Man4 to Man9. Error bars indicate mean ± SD (n = 3, "**" $P < 0.01$, "***" $P < 0.001$, "****" $P < 0.0001$ according to one-way ANOVA).

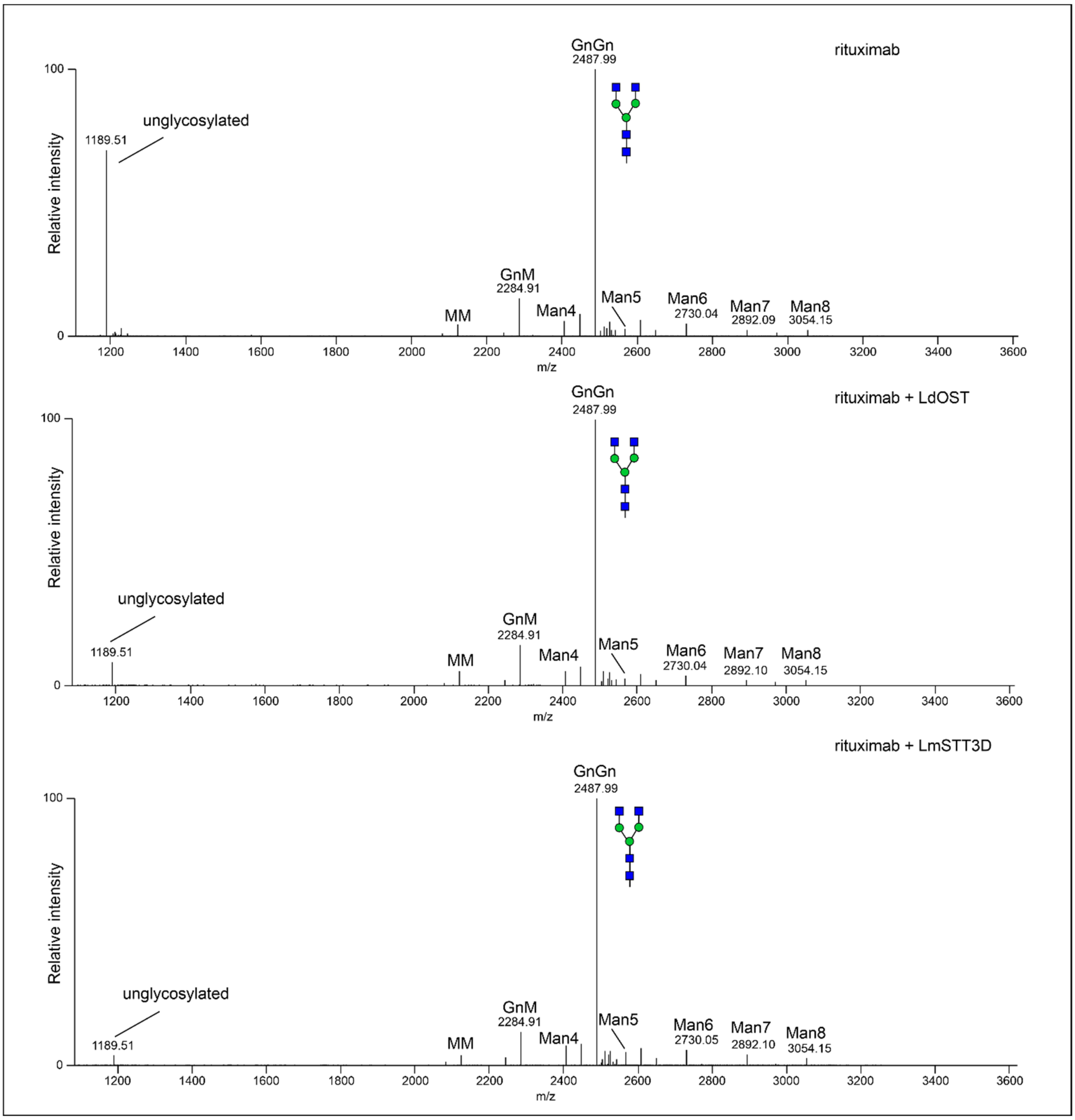

Figure S6. Representative MS-spectra of glycopeptides derived from rituximab expressed in the *N. benthamiana* ΔXF KO line. In the shown spectra, the peak at 1189.51 is assigned to the unglycosylated peptide EEQYNSTYR carrying Asn297, and the major peak at 2487.99 is assigned to the complex N-glycan GnGn (blue squares, GlcNAc, green cycles, mannose; nomenclature according to the ProGlycAn system: www.proglycan.com). No peaks corresponding to xylosylated or fucosylated N-glycans were detected.

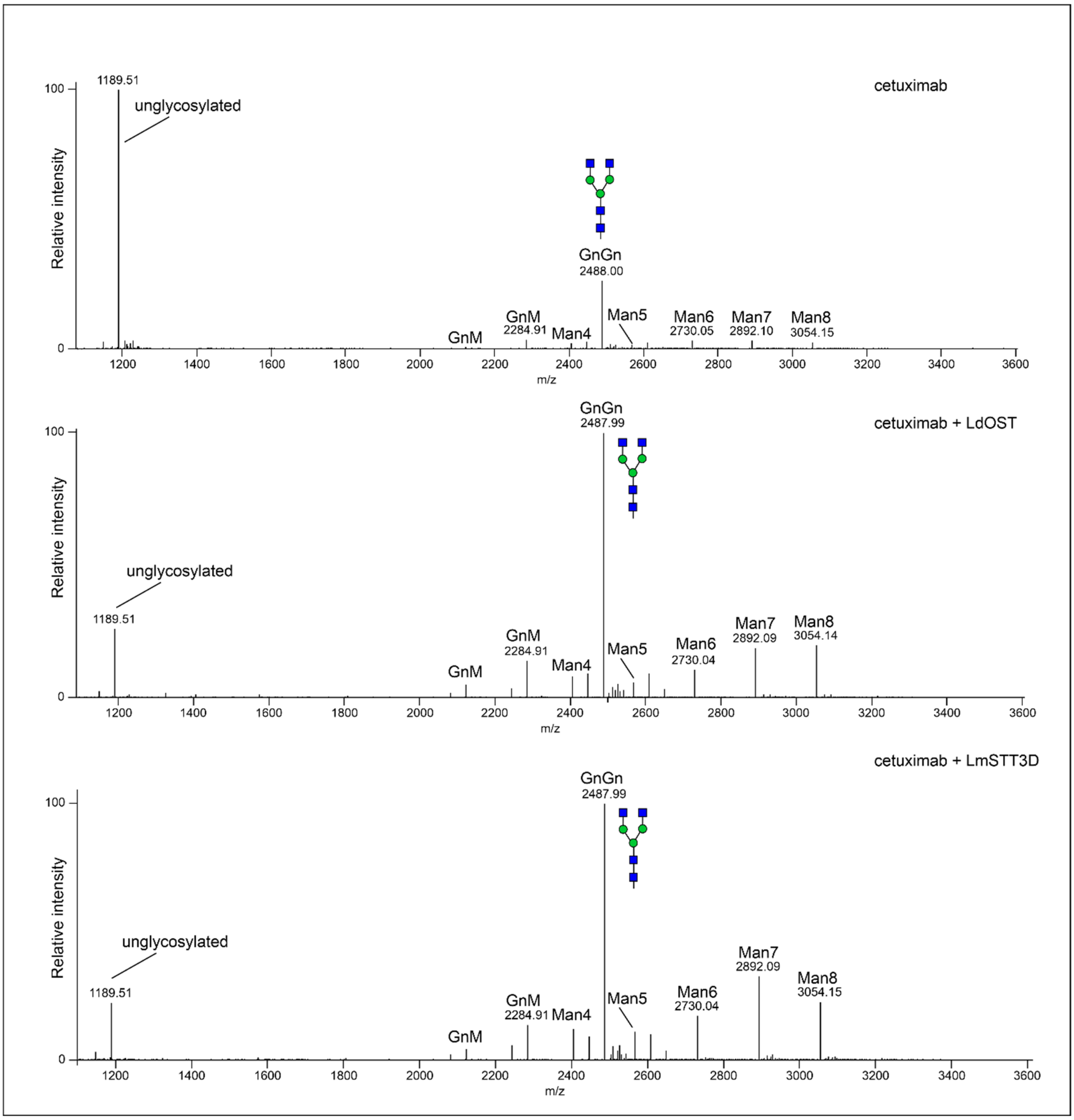

Figure S7. Representative MS-spectra of glycopeptides derived from cetuximab expressed in the *N. benthamiana* ΔXF KO line. In the shown spectra, the peak at 1189.51 is assigned to the unglycosylated peptide EEQYNSTYR carrying Asn297, and the major peak at 2487.99/2488.0 is assigned to the complex N-glycan GnGn (blue squares, GlcNAc, green cycles, mannose; nomenclature according to the ProGlycAn system: www.proglycan.com). No peaks corresponding to xylosylated or fucosylated N-glycans were detected.

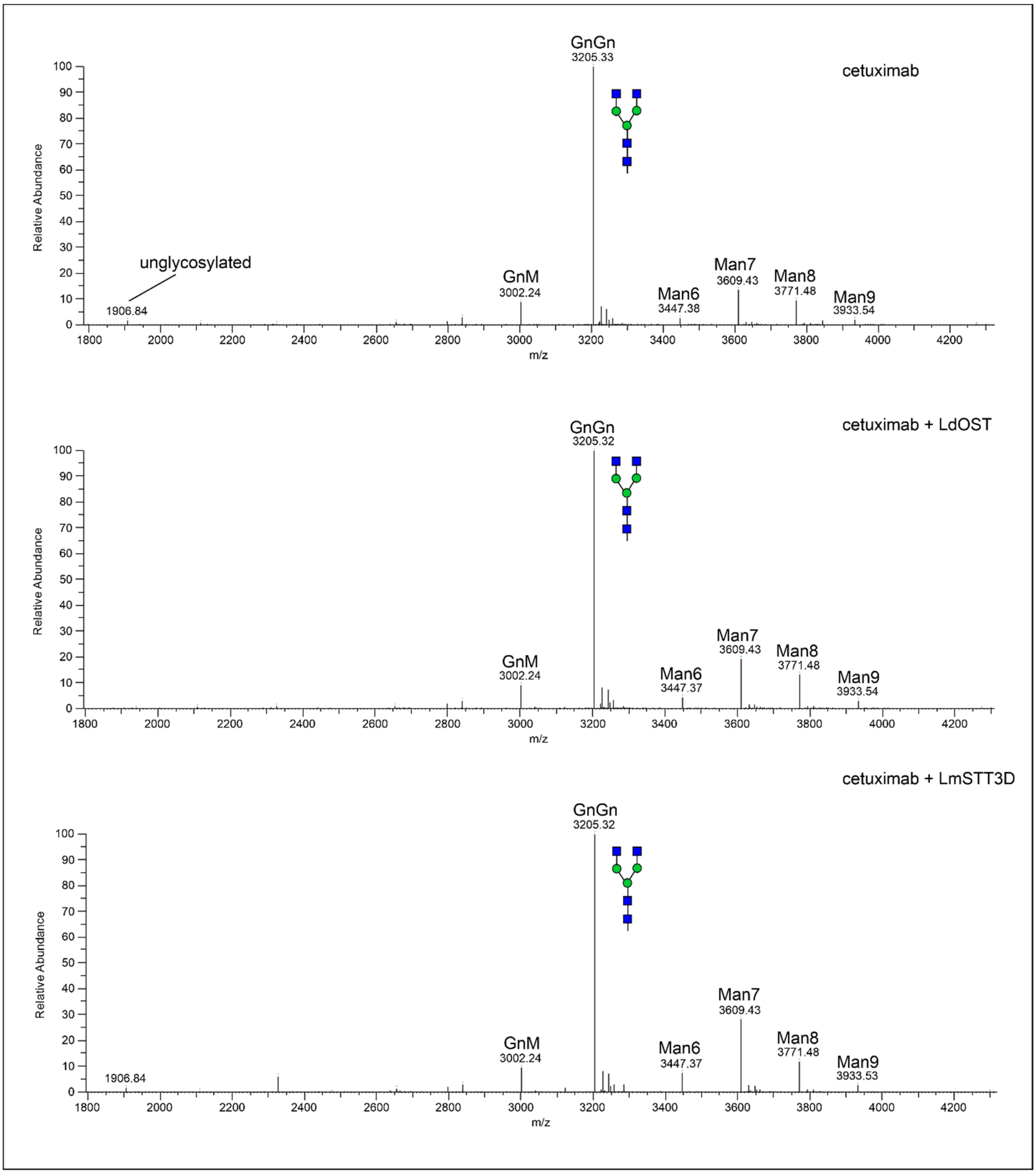

Figure S8. Representative MS-spectra of glycopeptides derived from the variable region of cetuximab expressed in the *N. benthamiana* ΔXF KO line. In the shown spectra, the peak at 1906.84 is assigned to the unglycosylated MNSLQSNDTAIYYCAR (Cys present as carbamidomethyl-cysteine) peptide, and the major peak at 3205.32/3205.33 is assigned to the complex N-glycan GnGn (blue squares, GlcNAc, green cycles, mannose; nomenclature according to the ProGlycAn system: www.proglycan.com). No peaks corresponding to xylosylated or fucosylated N-glycans were detected.

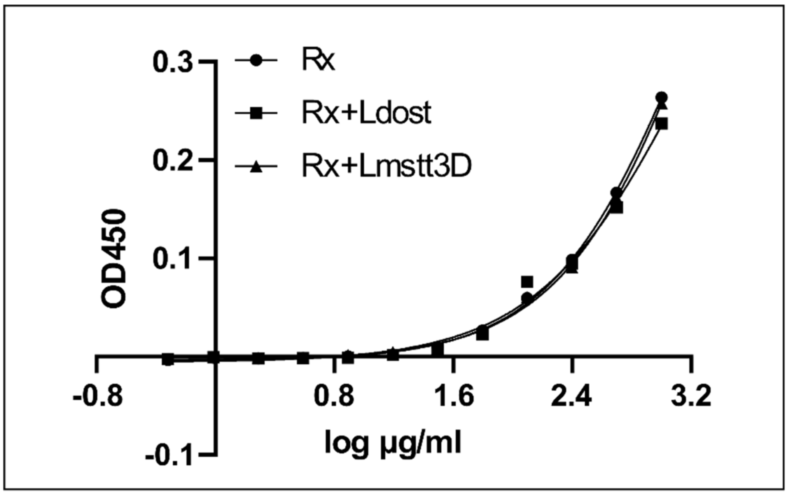

Figure S9. Antigen binding ELISA. Antigen binding ELISA of purified rituximab (Rx), rituximab co-expressed with LdOST (Rx+Ldost) and rituximab co-expressed with LmSTT3D (Rx+Lmstt3D).

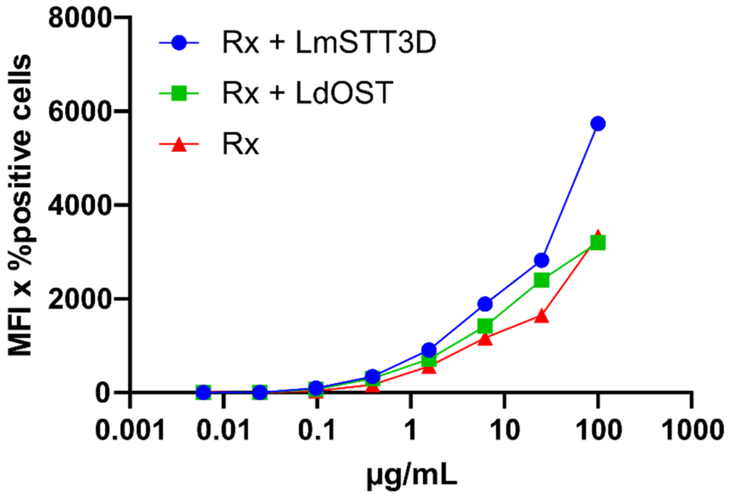

Figure S10. Cell-based receptor binding assay using TZM-bl FcγRIIIa (F158) cells. Rituximab (Rx) variant binding was determined by FACS analysis (Forthal et al., 2010). Instead of reporting only the percentage of positive cells or the median fluorescent intensity (MFI) alone an antibody binding score that represents quantity and intensity is shown at the Y-axis. All data points represent median binding values. No significant differences were observed between the Rx variants (based on ANOVA of the data set with the highest concentration followed by a multi-comparison Kruskal-Wallis-test between the groups).

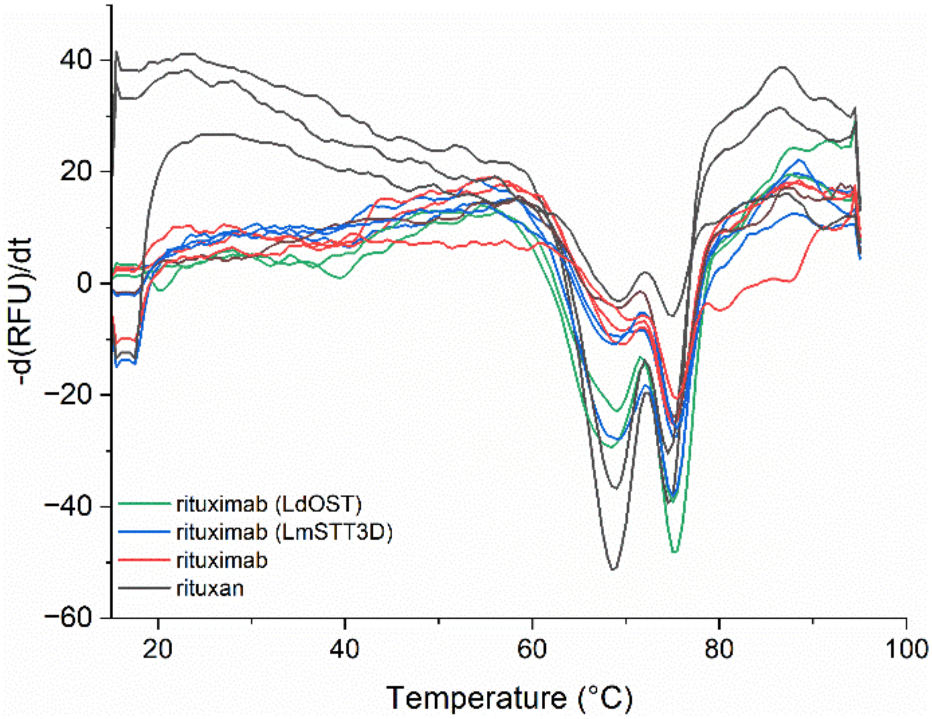

Figure S11. DSF profiles of the rituximab variants showing changes in protein intrinsic fluorescence with temperature. For each protein, overlaid curves (technical replicates) are shown.

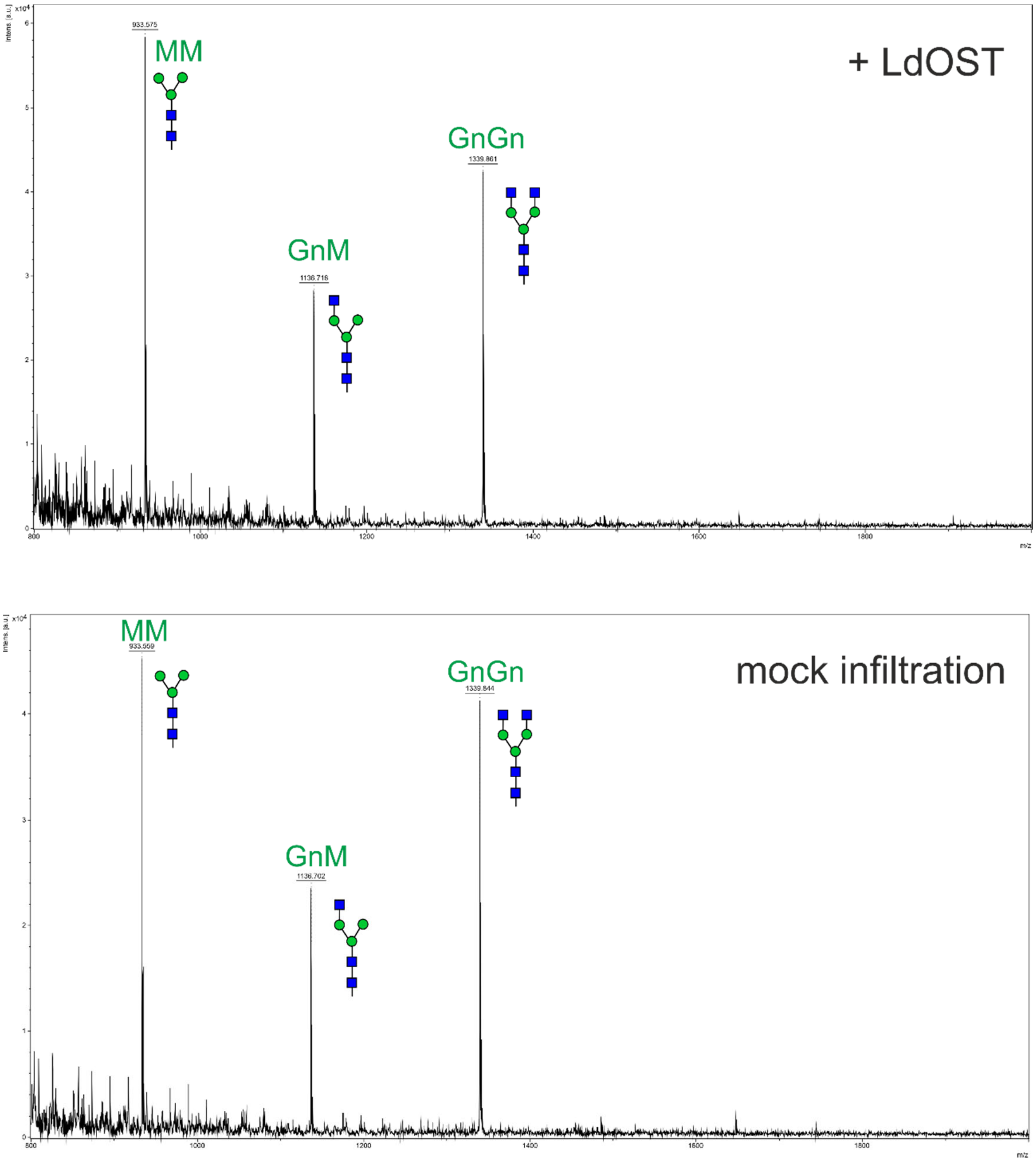

Figure S12. MALDI-MS analysis of N-glycans released from total soluble proteins (TSP). *N. benthamiana* ΔXT/FT plants (Strasser et al., 2008) were infiltrated with *Agrobacteria* suspensions (OD_{600} of 0.1) carrying either LdOST-TAG or p48-RBD (Shin et al., 2021) which was used as an unrelated control (mock infiltration). Formic acid (5% final concentration) and 40 µL pepsin (1 µg/µL) were added to 0.5 mL of TSP (in PBS buffer, pH 7.4) and incubated for 16 h at 37°C. After neutralization (pH 7.0) with 25% ammoniac, the mixture was passed over a C18 SPE clean-up column (elution with 80% acetonitrile/0.1% formic acid) and dried in a speed-vac. The dried samples were dissolved in 200 µL 50 mM ammonium acetate pH 5.5 and glycans were released from the peptides by incubation with PNGase A for 16 h at 37°C. After C18 clean-up the flowthrough was dried in a speed-vac, reconstituted in 20 µL H_2O and analyzed by MALDI (2,5-dihydroxybenzoic acid matrix, Bruker Autoflex Speed). Nomenclature of assigned peaks is according to the ProGlycAn system: www.proglycan.com), for an explanation of the symbols used for the illustration of glycans see legend of Fig. S6.

Table S1. SPR data. Association rate (ka), dissociation rate (kd), and kinetic dissociation constant (KD) values are shown.

		KD (nM)	ka ($M^{-1}s^{-1}$)	kd (s^{-1})
Human FcγRIIIa (V176)	Rituximab	18 ± 1	$6.60 \cdot 10^5$	$1.19 \cdot 10^2$
	Rituximab + LdOST	13 ± 1	$8.34 \cdot 10^5$	$1.12 \cdot 10^2$
	Rituximab + LmSTT3D	12.2 ± 0.8	$8.75 \cdot 10^5$	$1.07 \cdot 10^2$
	Rituxan	263 ± 38	-	-

		KD (nM)	ka ($M^{-1}s^{-1}$)	kd (s^{-1})
Human FcγRIIIa (F176)	Rituximab	66 ± 5	$7.09 \cdot 10^5$	$4.72 \cdot 10^2$
	Rituximab + LdOST	50.1 ± 0.2	$9.36 \cdot 10^5$	$4.69 \cdot 10^2$
	Rituximab + LmSTT3D	54 ± 1	$9.05 \cdot 10^5$	$4.91 \cdot 10^2$
	Rituxan	513 ± 205	-	-
